# Determinants of premenstrual dysphoric disorder and associated factors among regular undergraduate students at Hawassa University Southern, Ethiopia, 2023: institution-based cross-sectional study

**DOI:** 10.1186/s12889-024-18798-y

**Published:** 2024-05-23

**Authors:** Aklile Tsega Chekol, Yared Reta, Fikadu Ayinewa, Lielina Hailu, Mulualem Tesema, Mastewal Aschale Wale

**Affiliations:** Faculty of Health Sciences, College of Medicine and Health Sciences, Hawassa University, 1560, Hawassa, Ethiopia

**Keywords:** Premenstrual, Dysphoria, Menstruation, Undergraduate students, Hawassa University

## Abstract

**Background:**

Premenstrual dysphoric disorder (PMDD) is a condition causing severe emotional, physical, and behavioral symptoms before menstruation. It greatly hinders daily activities, affecting academic and interpersonal relationships. Attention is not given to premenstrual disorders among female students in higher education. As a result, students are susceptible to stress, and their academic success is influenced by various factors, including their menstrual cycle, and the long-term outcomes and consequences are poorly researched. Even though PMDD has a significant negative impact on student’s academic achievement and success limited research has been conducted in low- and middle-income countries including Ethiopia, especially in the study setting. Therefore, a study is needed to assess premenstrual dysphoric disorder and associated factors among regular undergraduate students at Hawassa University.

**Methods:**

An institutional-based cross-sectional study was conducted among 374 regular undergraduate female students at Hawassa University, College of Medicine and Health Sciences. A self-administered structured premenstrual symptoms screening tool for adolescents was used to assess premenstrual dysphoric disorder. The collected data were loaded into a statistical package for the social science version 25 and analyzed using it. Both bivariate and multivariate logistic regression were used to identify factors associated with premenstrual dysphoric disorder. Each independent variable was entered separately into bivariate analysis, and a variable with a *p*-value less than 0.25 were included in the multivariate analysis to adjust the possible confounders. Statistically significant was declared at a 95% confidence interval when variable with a *p*-value less than 0.05 in the multivariate analysis with premenstrual dysphoric disorder.

**Results:**

The magnitude of premenstrual dysphoric disorder in this study was 62.6% (95% CI 57.4–67.5). Having severe premenstrual pain (AOR = 6.44;95%CI 1.02–40.73), having irregular menstrual cycle (AOR = 2.21; 95% CI 1.32–3.70), students who had poor social support (AOR = 5.10;95%CI, (2.76–12.92) and moderate social support (AOR = 4.93;95%CI (2.18–11.18), and students who used contraception (AOR = 3.76;95%CI, 2.21-6,40) were statistically significant factors with the outcome variable.

**Conclusion:**

The prevalence of premenstrual dysphoric disorder was high as compared to other studies. There was a strong link between irregular menstrual cycle, severe menstrual pain (severe dysmenorrhea), poor social support, and contraception use with premenstrual dysphoric disorder. This needs early screening and intervention to prevent the complications and worsening of the symptoms that affect students’ academic performance by the institution.

## Background

Premenstrual refers to the period leading up to menstruation (the shedding of the uterine lining) in females [1]. Dysphoria is a state of profound unease, dissatisfaction, and unhappiness accompanied by physical symptoms like fatigue, change in appetite, difficulty in concentration, and disturbance in sleep [2] Premenstrual dysphoric disorder (PMDD) is a collection of physical, cognitive, and affective symptoms causing clinically significant distress or interference before the onset of menses, after which they become minimal or absent. The fifth version of the Diagnostic and Statistical Manual of Mental Disorders (DSM-5) states that the prevalence can range from 1.8 to 5.8% for a year, with possible increases from 13 to 18% [3]. Studies carried out globally have indicated that the prevalence of PMS is 40% in Europe, 85% in Africa, 46% in Asia, and 60% in South America [4].

Both the DSM-IV and DSM-5 diagnoses are based upon a premenstrual pattern of at least five physical, affective, and/or behavioral symptoms, with a requirement of at least one of the key affective symptoms of affective labiality (mood swings, tearfulness, sensitivity to rejection); irritability or anger that is often characterized by increased interpersonal conflicts; marked depressed mood, hopelessness, or self-deprecating thoughts; or anxiety, tension or feeling on edge. Women may also experience difficulty concentrating or a sense of feeling overwhelmed or out of control and cognitive-affective symptoms can be accompanied by behavioral and somatic symptoms such as loss of interest in usual activities, lack of energy, changes in appetite or food cravings, changes in sleep, and physical symptoms unique to the premenstrual such as breast tenderness, breast swelling or bloating [5]. At least one physical or psychological symptom is reported by 80% of women during the luteal phase of their menstrual cycle; nevertheless, the majority do not experience a severe impairment in their everyday lives (between 1.3% and 5.3%) [6].

The prevalence of moderate-to-severe premenstrual complaints ranges from 5 to 20% in women of reproductive age, globally and around 75% of all women of reproductive age may experience symptoms. PMS is characterized by one or more physical, emotional, or behavioral symptoms during the days before menstruation and was found in 94.8% of women of reproductive age (15 to 49 years) [7]. In its severe form, PMS has been linked to increased absenteeism from work and school, poor academic performance, high rates of suicidal thoughts and attempts, and acute mental health difficulties, even though the majority of women with the disease can carry out their daily activities [8, 9]. There was a statistically significant correlation between PMDD and dysmenorrhea [10–13]. According to various community-based studies, the global prevalence of PMDD among women ranged from 1.2 to 6.4% [14–16]. Research conducted in India revealed that 12.2% of students had PMDD, 67% did not want to attend school while they were menstruating, and 71% said they had trouble focusing when studying [17] Another study conducted in Nigeria likewise found that 36.1% of people had PMDD [18]. The prevalence of PMDD among health science students ranged from 13.8 to 72.5%, according to an Ethiopian study [10, 19–21].

Premenstrual symptoms affect up to 90% of women of childbearing age. However, only a smaller percentage of women match the criteria for premenstrual syndrome (PMS), and fewer than 10% of women are identified as having premenstrual dysphoric disorder (PMDD). Premenstrual symptoms are modest issues that may arise in many females, however premenstrual dysphoric disorder is a serious form of menstruation problem that shows somatic, bodily, and emotional liability [22].

Mood changes may be attributable to the effect estrogen and progesterone have on serotonin, γ-amino butyric acid, and dopamine are more sensitive to fluctuations in these sex hormones [6]. According to the complex pathophysiology of moderate to severe PMDD that predominantly involves central neurotransmitters, ovarian hormones, and neurosteroids, the main therapeutic approaches target both the brain neurotransmitter systems and the hypothalamus-pituitary-ovarian axis [23]. Premenstrual symptoms are distressing for up to 20% of reproductive-aged women and are associated with impairment in interpersonal, academic, and workplace functioning for at least 3–8%. It affected women who experienced almost 3000 days of severe symptoms during the reproductive years [24]. The burden of illness includes poorer sleep quality, daytime functioning, inattentiveness, remarkable impairment of academic performance, and absence from class. Furthermore, women diagnosed with PMDD may utilize more medical services, including visits to clinicians, prescription drugs, and over-the-counter treatments [25]. Students are particularly susceptible to the effects of stress on their physical and mental health and their success in school is impacted by economic, social, and other significant spheres of functioning, particularly before their period. The goal of medical education is to create highly skilled medical professionals who can lead the public health movement and provide excellent patient-centered care. Years of intense study and consistent practice are needed for this. Psychological discomfort may occasionally arise from students’ never-ending battle to become highly trained healthcare practitioners [26, 27]. Even though PMDD has a significant negative impact on student’s academic achievement and success attention isn’t given and limited research has been conducted in low- and middle-income countries including Ethiopia, especially in the study setting. Therefore, a study is needed to assess premenstrual dysphoric disorder and associated factors among regular undergraduate students at Hawassa University.

## Methods and materials

### Study area, period, and design

An institutional-based cross-sectional study was conducted from September 5 to October 8, 2023, at Hawassa University College of Medicine and Health Sciences. It is far from Addis Ababa by 275 km, the capital city of the country. Hawassa University Referral Hospital is currently educating about 628 females in undergraduate programs in 12 departments. The number of students in each department is as follows; in public health school there are 30 students, in the school of medicine there are 238 students, in the school of nursing there are 69 students, medical laboratory 25 students, midwifery 45 students, optometry 34 students, environmental health 33 students, anesthesia 24 students, radiology 51 students, pharmacy 43 students, and health informatics 18 students.

### Study population

All regular undergraduate female students at Hawassa University, College of Medicine and Health Sciences were the source population. All selected regular undergraduate female students at Hawassa University, College of Medicine and Health Sciences were the study population.

### Inclusion and exclusion criteria

All regular undergraduate students at Hawassa University Referral Hospital with the age of 18 and above were included in the study while, a student who was ill during data collection and students who were not found during data collection time were excluded from the study.

### Sample size determination and procedure

The sample size was determined by using a single proportional formula under the following assumptions; a proportion of 66.9% from a previous study in Wollo University [28] with a 5% margin of error and 95% CI.

$${\text{n}} = \frac{({\text{Z}}_{\alpha/2})^2 {\text{p}} (1-{\text{p}})}{{\text{d}}^2}, {\text{n}} =\frac{(1.96)^2[0.669(1-0.669)]}{(0.05)^2}=340$$

The final calculated sample size was 374 including a 10% non-response rate.

**Fig. 1:**
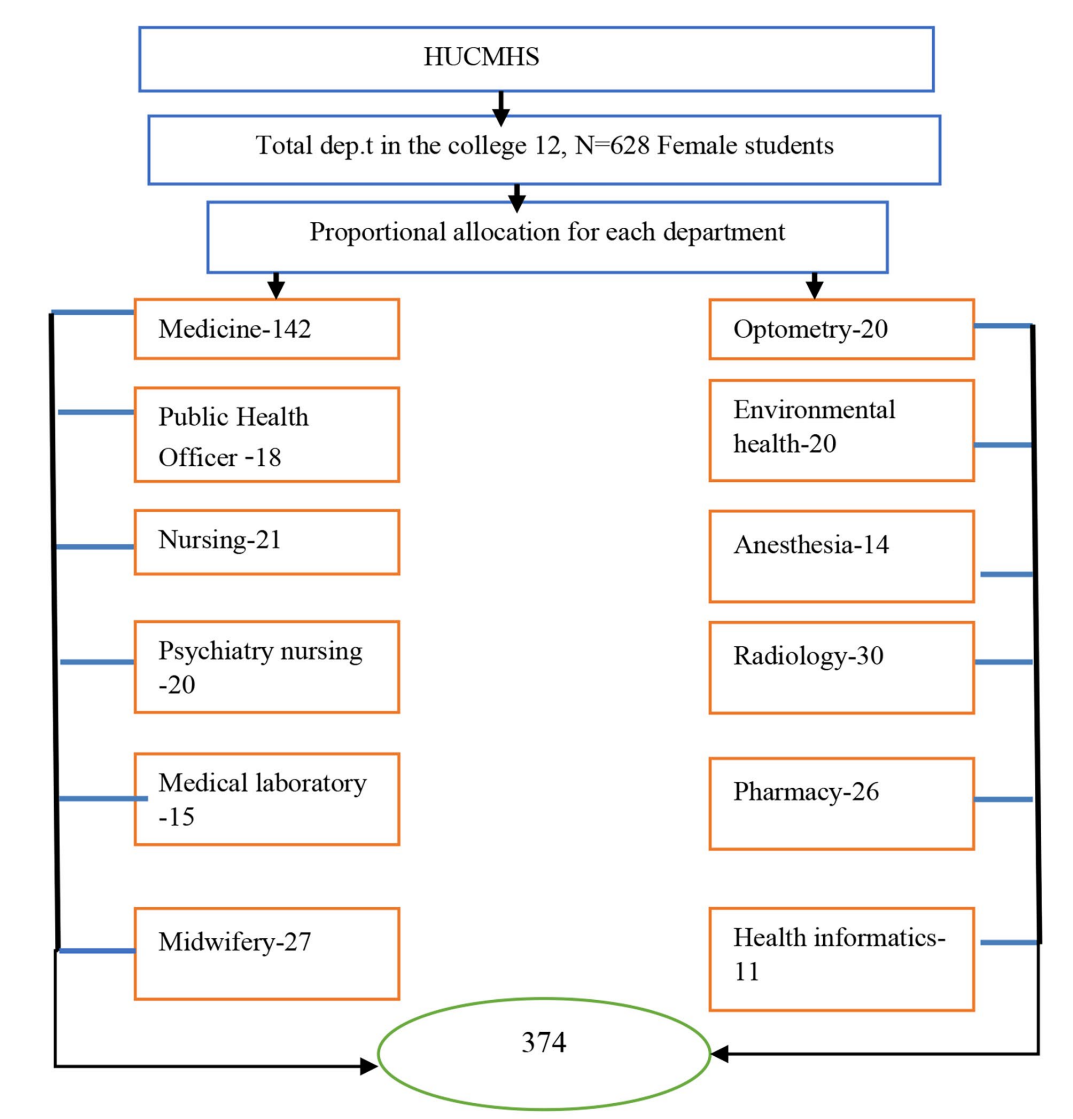
Proportionate allocation of stratified random sampling method to be used to assess the prevalence and associated factors of PMDD among Hawassa University referral hospital students, Hawassa, Ethiopia,2023

At first, a stratified random sampling method was used for each department. The entire sample was distributed by each department’s population size. Finally, a computer-generated simple random sampling method was used to select the study participants using their sampling frame that was obtained from the registrar. The sampling frame contains students’ identification number, student’s department, and students’ CGPA (Fig. 1).

### Data collection tools

The data was collected by three BSc Psychiatry nursing professionals using a semi-structured self-administered questionnaire supervised by two mental health specialists. The questionnaire encompassed seven basic sections. The first part was the socio-demographic characteristics of the study participants. The second section contains a premenstrual symptom screening tool for adolescents (PSST-A). The questionnaire was used to assess the severity of premenstrual symptoms. It is 19 item question with a four-point Likert scale (not at all = 0, mild = 1, moderate = 2, and severe = 3) and has two categories: the first 14 items assess physical, psychological, and behavioral symptoms, and the final five items are assessed the impact of symptoms on the life of women. The level of severity is determined using the sum of PSST scores and classified as; mild (0–19), moderate, and severe > 28 [29, 30]. The PSST states that to be classified as positive for PMDD, a person must meet three requirements: (1) have at least five symptoms from the first domain, rated as moderate to severe; (2) have at least one of the first four core symptoms (furious/angry; anxious/tensed; tearful/increased sensitivity to rejections; and depressed mood/hopelessness) rated as severe; and (3) have a significant functional impact of approved premenstrual manifestations [31]. Premenstrual psychological and physical symptoms are listed on the PSST, along with a functional impairment score based on DSM-5 criteria. It has excellent internal consistency and 0.91 content validity characterizing the PSST for teenagers above the age of 18 [32]. The correlation matrix for the 19 items was done. The data met the following criteria: KMO (0.556 approximately 0.6), Bartlett’s test of sphericity (χ2 _171_ = 458.914, *P* < 0.0001), and eigenvalues > 1.0 for the appropriateness of the model. The third section contains academic demand variables, which is a five-item structured questionnaire that assesses students’ interest in their field of study and the impacts of student’s interest on their academic performance and is also used to assess academic stress of their field of study. The fourth section contains health-related variables which is a 7-item questionnaire, recorded by structured yes/no responses. The questionnaire was designed to assess a diagnosed mental illness (e.g., depression, anxiety, and other mental illnesses) and chronic medical illnesses (e.g., Diabetes mellitus and Hypertension) in the respondents and the respondent’s family. The fifth section contains obstetrics and gynecology factors which is a 6-item questionnaire designed to assess the regularity of the menstruation cycle, menstrual flow during one cycle, and the level of menstrual pain during menstruation. The sixth section contains the psychosocial-related factor assessed by the Oslo-3 social support scale which ranges from 3 to 14. Classified as poor social support (3–8), moderate social support (9–11), and strong (12–14) [33]. The last section contains substance-related factors assessed by structured yes/no questions.

### Data quality control

The structured questionnaire was prepared in English and a brief explanation was given to the data collector about data collection tools and procedures, and how to keep the confidentiality of the participants during the training before the actual data collection period. Each completed questionnaire was evaluated by the principal investigator, and appropriate feedback was sent the next day. A pretest was done on 5% (19) of the sample size at Hawassa University’s main campus before conducting the main study to assess the clarity, sequence, consistency, and understandability of the questionnaires (α = 0.79). The outcome of the pretest was left out of the primary research. Based on the results of the pretest, the final data-gathering instrument was improved. The goal, method, advantages, and risks of the study were explained to the respondents, who were also given assurances regarding the privacy of their personal information. Additionally, participants had the right to withdraw from the study at any point during the interview if they felt uncomfortable.

**Data processing and analysis**.

After the data were verified to be accurate and consistent, they were coded. The coded data were loaded into a statistical package for the Social Science version 25 and analyzed using it. Descriptive statistics was used in frequency, mean, median, tables, text, and graphs to explore the characteristics of study subjects. Both bivariate and multivariate logistic regression were used to identify factors associated with premenstrual dysphoric disorder. Each independent variable was entered separately into bivariate analysis, and a variable with a *p*-value less than 0.25 were included in the multivariate analysis to adjust the possible confounders. Statistically significant was declared at a 95% confidence interval when variable with a *p*-value less than 0.05 in the multivariate analysis with premenstrual dysphoric disorder.

### Operational definition

The premenstrual symptom screening tool for adolescents is a standard tool to identify PMDD and the sum scores of PSST > 28 showed having PMDD [29].

Social support: Measured by the Oslo-3 social support scale which ranges from 3 to 14. A score (3–8) represents poor social support (3–8), (9–11) moderate social support, and (12–14) strong social support [33].

## Results

### Socio-demographic characteristics of respondents

Among the total number of 374 distributed questionnaires, all were filled completely and consistently and made a response rate of 100%. The majority of study participants 345 (92.2%) were found in the age group of 19–25 years. Regarding current residence majority 326(87.2%) of the participants live in the dormitory and (86.9%) of the participants’ families live by a distance of less than or equal to five hundred kilometers. Regarding marital status majority 341(91.2%) of the participants were single (Table 1).

**Table 1:** Socio-demographic characteristics of regular undergraduate students at Hawassa University Referral Hospital, College of Medicine and Health Sciences Hawassa, Southern Ethiopia (*n* = 374)

Variables	Categories	Frequency	Percentage
Age [34, 35]	19–25	345	92.2
	> 26	29	7.8
Religion	Orthodox	151	40.4
	Protestant	124	33.2
	Islam	68	18.2
	Catholic	17	4.5
	Other	14	3.7
Current Residency	Lives in dormitory	326	87.2
	In rent house	16	4.2
	Lives with family	32	8.6
Distance from a place where the family lives (KM)	< 500	325	86.9
	> 500	49	13.1
Family size in the house	< 5	170	45.5
	> 5	204	54.5
Parental marital status	Married	358	95.8
	Widowed	8	2.1
	Divorced	8	2.1
Student marital status	Single	341	91.2
	In relationship	23	6.1
	Married	10	2.7
Occupation of father	Government employee	117	31.3
	Merchant	102	27.3
	Private employee	89	23.8
	Other	66	17.6
Occupation of mother	Housewife	119	31.9
	Government employee	76	20.3
	Merchant	116	31.0
	Private employee	42	11.2
	Other	21	5.6
The educational status of the father	College and above	203	54.3
	9–12 Grade	109	29.1
	1–8 Grade	33	8.8
	Able to read and write	24	6.4
	Unable to read and write	5	1.3
Educational status of the mother	College and above	128	34.2
	9–12 Grade	127	34.0
	1–8 Grade	47	12.6
	Able to read and write	59	15.7
	Unable to read and write	13	3.5
Student’s average monthly pocket money in Ethiopian birr	< 1,000	115	30.7
	1,000–2,000	114	41.2
	> 2,000	105	28.1
The monthly income of the family in Ethiopian Birr	8,900 − 39,900	122	32.6
	2,250-8,900	240	64.2
	< 2,250	12	3.2

### Academic related factors

Of the study participants, one-third 125(33.4%) were fourth-year students. Concerning the field of study more than one-third 142(38.0%) were Medicine students. More than two-thirds 257(68.7%) of the participants were interested in the field of study (Table 2).

**Table 2:** Distribution of academic demand variables of respondents among regular undergraduate students at Hawassa University Referral Hospital, College of Medicine and Health Sciences Hawassa, Southern Ethiopia, 2023 (*n* = 374)

Variables	Categories	Frequency	Percentage
Year of Study	2nd year	72	19.3
	3rd year	89	23.8
	4th year	125	33.4
	5th year and above	88	23.5
Field of study	Anesthesia	14	3.7
	Environmental Health	20	5.3
	Public health	18	4.8
	Medical radiology	30	8.0
	Pharmacy	26	7.0
	Health informatics	11	2.9
	Medicine	142	38.0
	Midwifery	27	7.3
	Optometry	20	5.4
	Psychiatry nursing	30	8.0
	Nursing	21	5.6
	Medical laboratory	15	4.0
Interest in the field of study	Interested	257	68.7
	Not interested	117	31.3
Academic stress	No	101	27.0
	Low	24	6.4
	Medium	197	52.7
	Strong	52	13.9
Student’s previous cumulative grade (CGPA)	< 3	25	6.7
	3-3.5	227	60.7
	> 3.5	122	32.6

### Clinical and psychological-related factors

Out of 374 participants, the majority 349(93.3%) had no history of sexual abuse. Concerning daily sleep hours more than half 203(54.3%) of the participants arranged their sleep hours greater than 7 h. About having known chronic medical illness majority (88.5%) have no known chronic medical illness (Table 3).

**Table 3:** Distribution of health-related variables among regular undergraduates at Hawassa University Referral Hospital, College of Medicine and Health Sciences Hawassa, Southern Ethiopia, 2023 (*n* = 374)

Variables	Categories	Frequency	Percentage
History of sexual abuse	Yes	25	6.7
	No	349	93.3
History of emotional abuse	Yes	85	22.7
	No	289	77.3
History of depression	Yes	22	5.9
	No	352	94.1
History of mental illness other than depression	Yes	13	3.5
	No	361	96.5
Daily sleep hour	< 2	2	0.5
	2–4	52	13.9
	5–7	117	31.3
	> 7	203	54.3
History of known chronic medical illness	Yes	43	11.5
	No	331	88.5
Family history of medical illness	Yes	129	34.5
	No	245	65.5

### Obstetrics and gynecological-related factors

The monthly interval of the menstrual cycle for the majority of 221 (59.1%) participants was regular and the amount of bleeding (menstrual flow) during one cycle as reported by the respondents was minimal by 260 (69.5%). The majority of the participants 302(80.5%) had moderate to severe dysmenorrhea (pain during menstruation). Concerning contraception use more than half 215(57.5%) of respondents have not used contraception (Table 4).

**Table 4:** Obstetrics and gynecologically related variables among regular undergraduate students at Hawassa University Referral Hospital, College of Medicine and Health Sciences Hawassa, Southern Ethiopia, 2023 (*n* = 374)

Variables	Category	Frequency	Percentage
Regular menstruation	Yes	221	59.1
	No	153	40.9
Menstrual flow during one cycle	Minimal	260	69.5
	Moderate	27	7.2
	Heavy	87	23.3
Menstrual pain and its level	No	61	16.3
	Minimal	11	2.9
	Moderate	188	50.3
	Severe	114	30.5
Contraception use	Yes	159	42.5
	No	215	57.5
Age at first menses	< 13	197	52.7
	13–16	172	46.0
	> 16	5	1.3
Family history of dysmenorrhea	No	28	7.5
	Minimal	70	18.7
	Moderate	177	47.3
	Severe	99	26.5

### Psychosocial-related factors

**Fig. 2:**
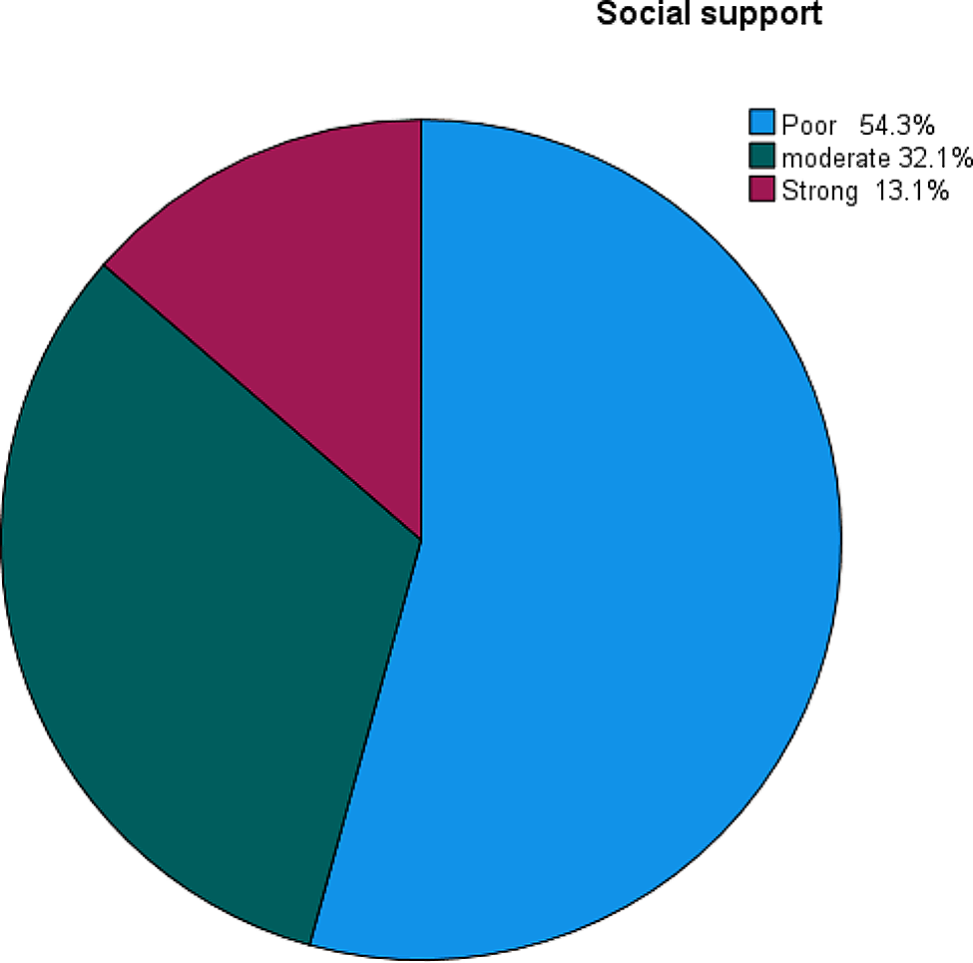
Level of social support among regular undergraduate students at Hawassa University Referral Hospital, College of Medicine and Health Sciences Hawassa, Southern Ethiopia, 2023 (*n* = 374)

Out of 374 participants, more than half of 203(54.3%) had poor social support while nearly one-third of 120(32.1%) participants had moderate and the rest 51(13.1%) had strong social support (Fig. 2).

**Behavioral-related factors**.

**Fig. 3:**
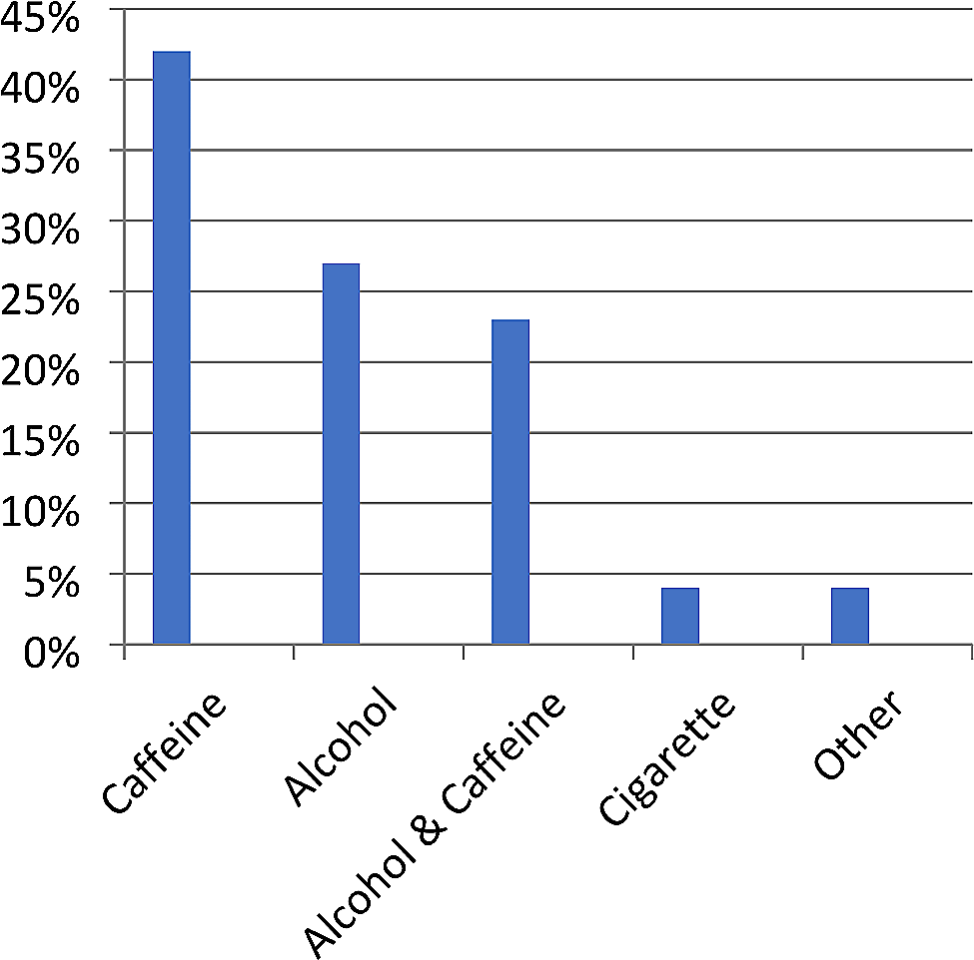
Behavioral-related factors among regular undergraduate students at Hawassa University Referral Hospital, College of Medicine and Health Sciences Hawassa, Southern Ethiopia, 2023(*n* = 374)

Of the total participants more than one-third 130(34.8%) of were used substances during their lifetime. Of these, more than two-fifths used caffeine. More than one-fifth 107(28.6%) of the study participants used alcohol. 90(24.7%) of the study participants used substances in the past 3 months and the most used substances were alcohol and caffeine. Of the participants, 39(10.4%) used those substances monthly (Fig. 3).

### The prevalence of PMDD

**Fig. 4:**
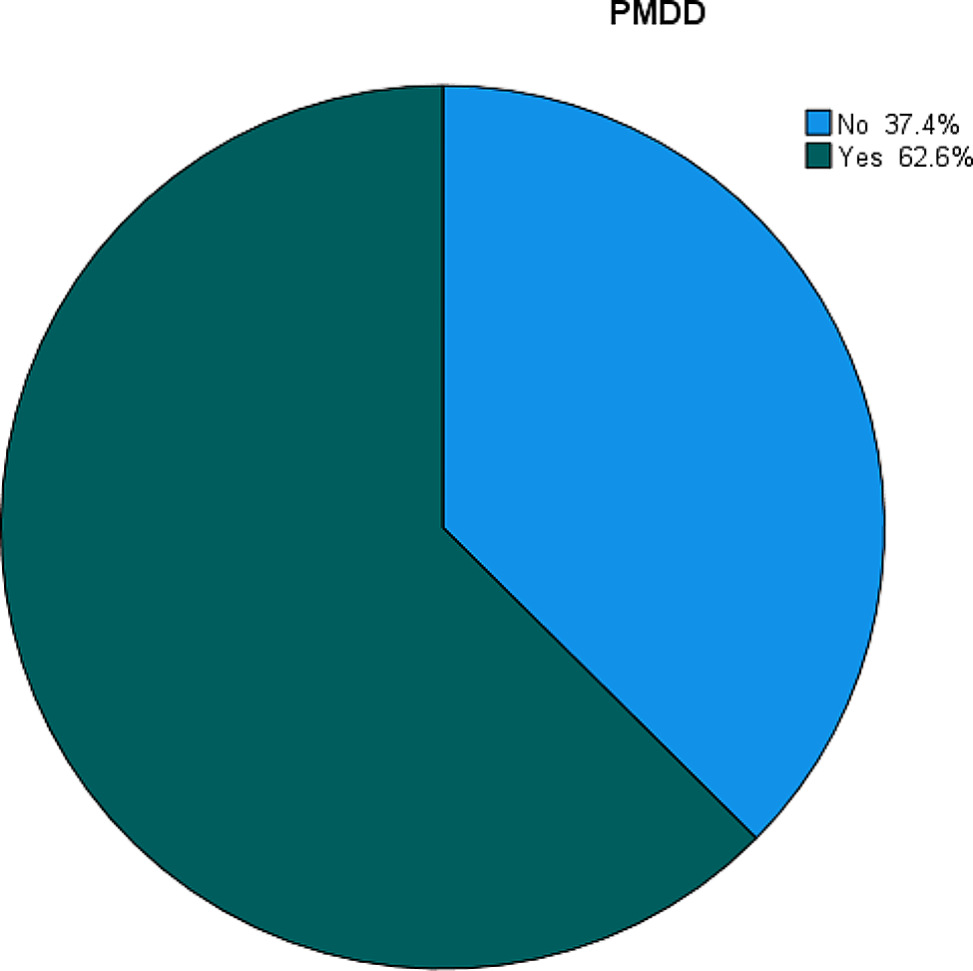
Prevalence of premenstrual dysphoric disorder among regular undergraduate students at Hawassa University Referral Hospital, College of Medicine and Health Sciences Hawassa, Southern Ethiopia, 2023 (*n* = 374)

The overall prevalence of PMDD in this study was 62.9% (95% CI 57.4–67.5). Based on the severity scale 0.8% of the participants had a mild form of PMS, and 36.6% of the participants had a moderate form of PMS (Fig. 4).

### Factors associated with PMDD

Both bivariate and multivariate logistic regression analyses were done to identify factors associated with the premenstrual dysphoric disorder. Each independent variable was entered separately into the bivariate analysis, and a variable with a *p*-value less than 0.25 was included in the multivariate analysis. During analysis on bivariate logistic regression age, interest in the field of study, regularity of menstrual cycle, pain during menstrual, social support, contraception use, and family history of severe discomfort during menstruation had a *p*-value of less than 0.25. All these variables were added to the final model of multivariate logistic regression to adjust the possible confounders. In this model, only irregular menstrual cycle, severe menstrual pain (severe dysmenorrhea), poor social support and contraception use were statistically significant with the outcome variable.

Students who had severe pain during menses were sixfold increased to develop PMDD as compared to those who had no pain (AOR = 6.44; 95% CI 1.02–40.73). In this study, students who had irregular menses were two times more likely to develop PMDD compared with those who had regular menstruation (AOR = 2.21; 95% CI 1.32–3.70). In the current study, students who had poor social support were sixfold more likely to develop PMDD compared to those who had strong social support (AOR = 5.97; 95% CI 2.76–12.92) while students with moderate social support were fivefold increased to develop PMDD compared with those who had strong social support (AOR = 4.93; 95% CI 2.18–11.18). In this study, those students who used contraception were fourfold more likely to develop PMDD when compared with those who didn’t use contraception (AOR = 3.76;95% CI 2.21–6.39) (Table 5).

**Table 5:** Bivariate and multivariate logistic regression analysis and associated factors with PMDD among regular undergraduate students at Hawassa University Referral Hospital, College of Medicine and Health Sciences Hawassa, Southern Ethiopia, 2023 (*n* = 374)

Variables	Categories	*PMDD*		COR (95%CI)	AOR (95%CI)	*P*-value
		Yes	No			
Age	19–25	220	125	1.89(0.88–4.04)	1.92(0.81–4.57)	0.139
	> 26	14	15	1	1	
Regularity of menses	Regular	121	100	1	1	
	Not regular	113	40	2.34(1.49–3.56) *	2.21(1.32–3.70) **	**0.003**
Menstrual pain (dysmenorrhea) and it’s level	No	28	33	1	1	
	Minimal	9	2	3.02(1.58–5.78)	2.13(0.99–4.61)	0.055
	Moderate	115	73	1.86(1.04–3.33)	1.52(0.76–3.02)	0.237
	Sever	82	32	5.34(1.06–26.61) *	6.44(1.02–40.73) **	**0.048**
Social support	Poor	141	62	6.01(3.03–11.91) *	5.97(2.76–12.92) **	**< 0.001**
	Moderate	79	41	5.09(2.48–10.48) *	4.933(2.18–11.18) **	**< 0.001**
	Strong	14	37	1	1	
Family history of severe discomfort during menstruation	No	15	13	1	1	
	Minimal	55	15	1.33(0.57–3.10)	2.80(0.89–8.84)	0.079
	Moderate	104	73	1.24(0.55–2.75)	1.04(0.40–2.79)	0.931
	Severe	60	39	3.18(1.25–8.11)	1.01(0.36–2.84)	0.991
Interest in the field of study	Interested	147	110	1	1	
	Not interested	87	30	2.17(1.34–3.52)	1.70(0.96–2.97)	0.067
Contraception use	Yes	123	36	3.21(2.03–5.06) *	3.76(2.21–6.39) **	**< 0.001**
	No	111	104	1	1	

**=<0.05, *<0.25, AOR = Adjusted Odds Ratio; COR = Crude Odds Ratio; CI = Confidence Interval;1 = Reference group

## Discussion

Even though menstruation is a natural phenomenon there were physical, physiological, and psychological problems associated with their day-to-day activities. Most women’s premenstrual symptoms can be eased or lessened with lifestyle modifications including eating less carbs and increasing exercise, as well as medication therapy using hormones or psychiatric medications. Serotonin reuptake inhibitors are now the first-choice medication for treating premenstrual dystrophic conditions. Academic performance is negatively impacted by PMDD symptoms in female students [36, 37]. As a result, it’s important to find out how common PMDD is and what factors are linked to it. Also, this would aid in problem prevention and the development of treatment plans that support female university students’ academic success.

The prevalence of premenstrual dysphoric disorder among regular undergraduate students at Hawassa University College of Medicine and Health Sciences was 62.6% (95% CI 57.4–67.5). This finding is similar to the studies conducted in Wollo University Health Sciences 66.9% [10] and 64.6% in Japan [38].

The results of the study conducted in Islamabad; Pakistan showed 81.2% which is higher than the current study [11, 39]. Furthermore, a study carried out in Iran revealed 98.2% had PMS which is greater than the results of the current study [40], also studies carried out in Debre Markos town [41] and Addis Ketema preparatory school [42] revealed that 81.3% and 86.1%, respectively, which is higher than the current study finding. The difference could be due to the difference in sample size, and measuring tools. An investigation carried out in Islamabad Pakistan employed a 22-item, structured questionnaire with 224 participants by using ICD-10 criteria [39]. There were 300 participants in a study conducted at Zahedan University in Iran using the DSM-IV diagnostic criteria [40]. Studies from 496 to 210 participants, respectively, in Debre Markos Town and Addis Ketema Preparatory School were done using the American College of Obstetricians and Gynecologists’ criteria. The degree of disease behavior and seeking medical attention, cultural and psychosocial factors also seem to affect the ratio and intensity of physical versus behavioral symptoms for PMS [43]. However, 374 participants in the current study were assessed with the PSST-A. The other rationale could be female university students view academic life as demanding and more likely to cause psychological disorders because they are always aiming for higher academic results [44, 45].

The present study’s finding, however, is greater than that of previous investigations. Jimma University [21], Mekelle University [46], University of Gonder College of Medicine and Health Sciences [47], Assosa Technical & Vocational Education College [20], Debre Berhan University [32], Egypt [48], and Nigeria [18] reported a prevalence of 27%, 37.0%, 34.7%, 26.8%, 49.3%, 21.1%, 36.1%, respectively. Furthermore, research from Baluchistan University in Iran [49–55], and Brazil [56] revealed 36.3% and 17.6%, respectively, which is less than the results of the current study. The possible reasons for this discrepancy could be data collection tools (DSM-5 and MINI) were used in the previous study while PSST-A was used in the current study [35]. Variations in the study population, geographic and cultural variance, and the various diagnostic standards and approaches all contributed to the prevalence of PMDD [17, 57]. Stressors of various intensities, societal perceptions of women’s roles and duties in college, and sociocultural elements are factors for the development of the problem [36].

### Variables associated with PMDD

In the final model of multivariate logistic regression irregular menses, pain during menstruation, use of contraceptives, and social support were statistically significant with PMDD.

The current study revealed that the severity of dysmenorrhea had a significant association with PMDD. Students who had severe pain during menstruation were more likely to experience PMDD as compared to students who had no menstrual pain (AOR = 6.44; 95% CI 1.02–40.73). This finding is supported by studies [10–13, 32, 54, 58–62]. Menstrual discomfort or dysmenorrhea aggravates emotional and behavioral reactions to menstrual symptoms that increase the risk of PMDD [47]. In addition, menstrual pain can heighten feelings of stress, anxiety, irritability, and sensitivity to rejection by others. This could be because the physiological effects of pain lead to mood swings, anxiety, appetite loss, cognitive decline, difficulty concentrating, impairment in work, and feelings of guilt about being female, which can lead to psychological symptoms and behavioral changes that can result in premenstrual syndrome [63]. To lessen the likelihood of related symptoms, which collectively result in premenstrual syndrome, students experiencing severe menstruation pain should seek medical attention and use non-pharmacological pain management techniques. In this study, irregular menstrual cycles were statistically significant with PMDD. Students who had irregular menses were two times more likely to develop PMDD compared with students who had regular menstruation (AOR = 2.207; CI 95% 1.318–3.697). This finding is in line with the study [54, 64, 65]. Possible explanations for this include fluctuating steroid hormones and unanticipated menstrual irregularities that occur without mental adjustment, which can cause psychological discomfort and related behavioral change [54, 64]. The finding is contradicted by Studies [66, 67]. The current study revealed that students who used contraception are four times more likely to develop PMDD when compared with those who don’t use contraception (AOR = 3.76;95% CI 2.21–6.40). This finding was contradicted by the studies [68, 69]. According to earlier research, women using oral contraceptives experience fewer and milder emotional symptoms overall, as well as fewer and milder physical symptoms [70–72]. Women with PMDD, a history of present or former mood or anxiety disorders, postpartum depression, sensitivity to progestins in the past, or a history of antidepressant usage should be identified by clinicians through a comprehensive medical history. A more comprehensive review of their past and present psychiatric histories should be conducted for those with risk factors [73]. Students who had poor social support were more likely to develop PMDD compared to those who had strong social support AOR = 5.972 95% CI (2.76–12.92) while students with moderate social support are five times more likely to develop PMDD compared with those who had strong social support (AOR = 4.93; 95% CI 2.18–11.18). There hasn’t been much research on the relationship between menstrual cycle symptoms and social support. Women with premenstrual depression (PMD) sought more social support premenstrual than women without PMD [74]. Thus, it stands to reason that premenstrual symptoms may be more common among women who believe they are receiving less assistance. The consequences of psychological characteristics like perfectionism and premenstrual distress can be mitigated by high levels of social support, which provides some protection [75]. Conversely, insufficient social support can worsen the unpleasant emotions, anger, and tension that come with the premenstrual period. It can also raise psychological anguish, feelings of isolation, and a lack of coping mechanisms. Knowing the psychological characteristics related to premenstrual symptoms experienced by the non-treatment-seeking population is valuable since some believe symptoms before menstruation are considered a female norm.

In this study, there was no statistically significant association between PMDD and any of the factors that were found to be significantly associated in previous studies [46, 48, 54, 76], including lower age at menarche, average length of one cycle of menstruation, and. family history.

Intervention tactics are to be tailored to each individual using different techniques and procedures that ought to be applied to address nutritional issues that fit women’s diverse needs and circumstances, as well as management factors that increase the problem. The results of this study should be actively used by educators, colleges, and other organizations to support women with PMDD.

### Strength and limitation

Only students from the College of Medicine and Health Sciences are included; this does not accurately reflect overall female university students. Since the subject is sensitive in our society, some people might be reluctant to discuss their actual personal issues.

There may be potential bias; arising from the use of a self-reporting questionnaire. The data was collected retrospectively due to financial constraints, which is the other limitation of the study, we recommended that the upcoming researcher conduct prospectively research considering with wider age.

## Conclusion

The prevalence of premenstrual dysphoric disorder was high as compared to other studies done in the country. Irregular menstrual cycle, severe menstrual pain (severe dysmenorrhea), social support, and contraception use had a significant association with PMDD. This needs early screening and intervention to prevent the complications and worsening of the symptoms that affect students’ academic performance in the institution.

### Clinical and public implications

It is better to start a stress-reduction program for female students that treats physical and psychological symptoms using both pharmacotherapy and non-pharmaceutical treatments. Medical professionals should develop and execute healthcare-seeking behavior in students since PMDD has a substantial detrimental impact on academic achievement as well as social and interpersonal interactions. Students with irregular menstrual cycles, severe dysmenorrhea (pain during menstruation), low social support, and contraception use should receive more attention. Providing social support and positive reframing should be taken as an intervention by health promotion programs for women with severe premenstrual syndrome. Support the health care team’s training in the diagnosis and treatment of PMDD for the benefit of the students by working with other stakeholders.

## Data Availability

The raw data may be available upon reasonable request by the corresponding author.
